# Multiple imputation is better than KDIGO guidelines for estimating unknown baseline renal function

**DOI:** 10.1186/s13054-016-1281-x

**Published:** 2016-04-15

**Authors:** Matthieu Jamme, Guillaume Geri

**Affiliations:** Intensive Care Unit, Cochin Hospital, Assistance Publique Hôpitaux de Paris, 27 rue du Faubourg Saint Jacques, 75014 Paris, France

We read with great interest the study of Wang et al. [1] recently published in *Critical Care* that assessed the impact of fluid balance on acute kidney injury (AKI) in critically ill patients. Fluid overload was independently associated with outcome in this large prospective Chinese cohort.

As recommended by the international Kidney Disease Improving Global Outcome guidelines, AKI was defined as an increase of serum creatinine (SCr) within 48 h from baseline SCr and/or urine output [2]. For patients without known baseline SCr, the KDIGO guidelines recommend using a hypothetical value of SCr assuming a “normal” estimated glomerular filtration rate (eGFR) of 75 mL/min/1.73 m^2^ [3]. This “simple imputation” could be an attractive method but tends to distort the distribution of variables and the association between them, which can lead to biased estimation [4].

Multiple imputation methods have been proposed and are nowadays considered one of the best methods for analyzing data sets with missing data values. Indeed, these methods adequately estimate the unknown parameters while biases have been found with single imputation methods. Unfortunately, multiple imputation methods are not frequently used in the intensive care unit literature. In the specific case of unknown baseline SCr in AKI studies, however, some authors have observed that multiple imputation methods had a lower rate of AKI misclassification compared with the “eGFR 75 simple imputation” [5].

We hope the authors will comment on their choice of imputation method.

## Abbreviations

AKI: acute kidney injury
eGFR: estimate glomerular filtration rate
KDIGO: Kidney Disease Improving Global Outcome
SCr: serum creatinine
