# Erratum to: Multiple imputation is better than KDIGO guidelines for estimating unknown baseline renal function

**DOI:** 10.1186/s13054-016-1317-2

**Published:** 2016-06-17

**Authors:** Matthieu Jamme, Guillaume Geri

**Affiliations:** Intensive Care Unit, Cochin Hospital, Assistance Publique Hôpitaux de Paris, 27 rue du Faubourg Saint Jacques, Paris, 75014 France

Unfortunately, the original version of this article [1] was publishing without its corresponding author response and associated references included. This has now been corrected in the original article.
